# Food Acquisition and Daily Life for U.S. Families with 4- to 8-Year-Old Children during COVID-19: Findings from a Nationally Representative Survey

**DOI:** 10.3390/ijerph18041734

**Published:** 2021-02-10

**Authors:** Mackenzie J. Ferrante, Juliana Goldsmith, Sara Tauriello, Leonard H. Epstein, Lucia A. Leone, Stephanie Anzman-Frasca

**Affiliations:** 1 Jacobs School of Medicine and Biomedical Sciences, University at Buffalo, Buffalo, NY 14214, USA; ferrant2@buffalo.edu (M.J.F.); jmgoldsm@buffalo.edu (J.G.); sarataur@buffalo.edu (S.T.); lhenet@buffalo.edu (L.H.E.); 2 Center for Ingestive Behavior Research, University at Buffalo, Buffalo, NY 14214, USA; 3 School of Public Health and Health Professions, University at Buffalo, Buffalo, NY 14214, USA; lucialeo@buffalo.edu

**Keywords:** COVID-19, families, children, food acquisition, restaurants

## Abstract

Evidence of short-term impacts of the coronavirus disease 2019 (COVID-19) pandemic on family life is emerging. Continued research can shed light on potential longer-term impacts. An online survey of U.S. parents with 4- to 8-year-old children (*n* = 1000) was administered in October 2020. The survey examined parent-reported impacts of COVID-19 on lifestyle (e.g., work, child-care, grocery shopping), as well as current family food acquisition and eating behaviors (e.g., cooking, restaurant use). Descriptive statistics were calculated, incorporating sampling weights based on sociodemographics. In terms of COVID-19 impacts, parents reported increases in working from home, decreased work hours, and increased child care and instruction, with most children attending school or receiving care at home. Parents reported increased home cooking and online grocery shopping; only 33% reported increased take-out or delivery from restaurants. About half of parents reported that their child dined at restaurants, 62% reported getting take-out, and 57% reported delivery from restaurants at least 2–3 times per month. About half viewed dining at restaurants as safe, while take-out and delivery were seen as safe by around three-quarters. Approximately two-thirds reported recent food insecurity. These nationally-representative results illustrate possible longer-lasting shifts in family life, with the potential to impact health and well-being. Sociodemographic differences and research and policy implications are discussed.

## 1. Background

The coronavirus disease 2019 (COVID-19) pandemic caused sudden and drastic lifestyle changes across the globe. In the United States (U.S.), protection measures were implemented beginning in March 2020 to halt the spread of the virus, closing restaurants, businesses, schools, and child-care facilities around the country and sending more than 50 million children back to their homes to finish the school year [1,2,3]. Families’ day-to-day lives were upended, as many suddenly needed to provide child care and schooling at home, often in combination with remote work or job loss as unemployment rates increased [4,5]. Evidence of short-term impacts of these drastic changes on energy-balance-related health behaviors, such as physical activity and screen time, in the early months of the pandemic is beginning to emerge. More than six months later, the COVID-19 pandemic continues, but in many regions, restaurants, businesses, and schools are now open under new restrictions [6]. Information on families’ behaviors under this “new normal” is limited. Examining families’ daily lives as the COVID-19 pandemic progresses is important, as potential new family routines and behaviors may have lasting positive, or negative, impacts on the health of families, as well as implications for research and policy.

Recently published survey data suggest that the early months of the pandemic brought changes in many energy-balance-related behaviors. In the initial months of the pandemic, there were reported increases in sedentary behavior and less time spent on physical activity for both adults and children [7,8,9]. This was coupled with increased screen time and sleep for children [7] and increased levels of stress and anxiety for adults, particularly parents [4,8,10]. In addition, almost 35% of households in the U.S. with children under the age of 18 indicated some form of food insecurity early in the pandemic, a substantial change from 11% in 2018/2019 [11,12,13]. Increased food insecurity early in the pandemic may be connected to increases in unemployment, which in the U.S. reached over 14% in April of 2020 with almost 19 million unemployment claims filed [5,14]. Food insecurity has been linked with unhealthy dietary and weight outcomes for children, highlighting that these impacts of COVID-19 have the potential to affect children’s eating and health in the long term [15,16].

Prior to the start of the pandemic, U.S. children had a notably poor diet quality, consumed few nutrient-dense foods such as whole grains, fruits, and vegetables, and frequently ate restaurant foods [17,18]. U.S. families spent a significant amount of their food dollars on restaurant foods and fewer food dollars on groceries and home cooked meals, a trend documented since the 1970s [19,20]. This trend in food spending translated to families eating out frequently and preparing less food at home [21,22], a trend that has also been observed across the world [23,24]. However, there is evidence that the pandemic may be reversing this trend. Surveys administered during stay-at-home orders indicated significant increases in meals prepared at home, and data from the Economic Research Service indicated an increase in food purchasing from retail stores [8,25]. Families also shifted where and how they shopped for food (e.g., altering the typical location of grocery trips and making more use of freezers and pantry staples) [10]. It is estimated that almost three-quarters of American households avoided restaurant food in the initial months of the pandemic, and household expenditures for restaurant foods were about 30% lower than they had been in March 2019 [13,25]. However, the initial months of the pandemic also brought increases in snacking and intake of foods such as sugar-sweetened beverages, potato chips, and red meat [7,9]. While results from the initial COVID-19 pandemic studies illustrate shifts in food acquisition and eating behavior, the extent to which these trends translate into longer-lasting changes for families is unknown. Research on family food acquisition and eating behavior during COVID-19 has primarily focused on the initial months of the pandemic and involved convenience samples. In addition, food acquisition from restaurants via take-out and delivery is understudied, both during COVID-19 and in general. Therefore, nationally representative studies examining families’ current behaviors related to food acquisition and restaurant use are warranted.

Taken together, emerging evidence suggests that the drastic lifestyle changes of the pandemic’s early months brought changes in energy-balance-related behaviors for children and families. However, little is known about whether these changes have persisted more than six months into the pandemic. A description of current behaviors can elucidate the extent to which COVID-19 may have longer-lasting impacts on families’ energy-balance-related behaviors and health. Therefore, in Fall 2020, we examined the following among a nationally representative sample of U.S. parents with at least one 4- to 8-year-old child: (1) parent-reported impacts of COVID-19 on various aspects of daily life, including food acquisition, physical activity, child care, and employment; and (2) families’ current food acquisition and eating behaviors, including preparation of meals at home, children’s consumption of restaurant foods in-person and via take-out and delivery, and factors affecting restaurant meal choices. 

## 2. Methods

### 2.1. Participants

Invitations to participate in this study were sent to a stratified random sample identified as U.S. residents 18 years of age or older with at least one 4- to 8-year-old child in the household (*n* = 1000). Participants were recruited using the Harris Poll Online opt-in panel (https://theharrispoll.com/), which includes millions of respondents who have agreed to participate in survey research. To be eligible, individuals needed to be English-speaking, at least 18 years of age, a parent/caregiver (referred to herein as parents) with at least one 4- to 8-year-old child, and have internet access. Possible participants were sent a password-protected email invitation to participate in the survey. 

### 2.2. Procedures

A 61-item survey was developed by researchers at the University at Buffalo in order to understand how parents with at least one 4- to 8-year-old child describe daily life and energy-balance-related behaviors during the COVID-19 pandemic. Harris Interactive was commissioned to disseminate the survey and incorporate sampling weights based on parent age, sex, race and ethnicity, education, income, region, marital status, household size, and number of children under 18 years, so results would be representative of the U.S. population of parents with 4- to 8-year-old children. Participating parents completed the survey at one time during October 2020. Participants with multiple 4- to 8-year-old children were asked to complete child-focused survey questions about their child with the most recent birthday. Study procedures were approved by the University at Buffalo Institutional Review Board. 

### 2.3. Measures

The survey was created using previously-developed measures from the existing literature as well as newly-developed items. Validated scales were used to measure perceived stress and food insecurity as described below. No changes were made to the former, while the latter’s time frame was modified to fit with the present study’s focus on the COVID-19 pandemic (i.e., specifying that responses should reflect experiences in the past two months, rather than the past year). Other survey items were adapted or created for this study as described herein. Overall, the main modification to existing items was to specify that responses should reflect experiences during the past two months where appropriate. Other minor modifications to existing items are described below.

#### 2.3.1. Participant Demographics and Context

Parents reported their age, gender, height, weight, marital status, highest level of education, household income, employment status, race/ethnicity, and whether the household received any government benefits (e.g., the Supplemental Nutrition Assistance Program (SNAP), which provides nutritional assistance to supplement food budgets, or Medicaid, which assists low-income individuals with health costs). Parents also reported characteristics of their 4-to-8-year-old child, including age, gender, race/ethnicity, and eligibility for free or reduced-price school meals. Additionally, they indicated who in the household was the most familiar with the child’s daily activities (responses included: I am, another parent/guardian, another parent/guardian and I are equally familiar). A brief validated 2-item screen was administered to identify households at risk for food insecurity [26], specifying that respondents should answer based on the past two months. Items assessed how often in the prior two months the household has ‘worried whether food would run out before we got money to buy more’ and ‘the food that we bought just didn’t last and we didn’t have money to get more.’ Responses included: often, sometimes, or never. Food insecurity is indicated when participants respond often or sometimes to at least one of the two items. Cronbach’s alpha for this 2-item screen in the present sample was 0.84. 

Parents were also asked a series of questions developed by the research team about the extent of current COVID-19 related protection measures in their town/city, including if mask wearing was mandated and whether there were restaurant-related restrictions. Children’s schooling and care in the last week (in-person elementary school, virtual elementary school, home school, and/or in- or out-of-home non-parental child care) was also assessed. Parental stress during the previous month was measured using the short version of the validated Perceived Stress Scale (PSS-4) [27]. Participants completed four questions, administered verbatim from the original scale, assessing the degree to which they perceived situations in their life to be stressful (e.g., ‘how often have you felt that you were unable to control the important things in your life?’). Items were rated on a five-point scale from never (0) to very often (4). Cronbach’s alpha for the PSS-4 in this sample was 0.56. The correlation between food insecurity and perceived stress scores was also examined as an indicator of convergent validity and was 0.31 (*p* < 0.0001).

#### 2.3.2. Daily Life Changes during the Coronavirus Disease 2019 (COVID-19) Pandemic 

Parent-reported changes in daily life and energy-balance-related behaviors were assessed using questions adapted from The Epidemic–Pandemic Impacts Inventory (EPII), establishing who in the household was affected by changes related to care of, instruction of, and quality time with children, and care for other family members [28]. Parents were also asked whether or not (yes or no) their work had been affected in a variety of ways, including job loss and increased or decreased work hours, using a question and response options from the National Institute of Health’s Environmental influences on Child Health Outcomes (ECHO) questionnaire [29]. The extent to which the COVID-19 pandemic resulted in changes in various lifestyle behaviors was also assessed using questions adapted from the ECHO questionnaire [29]. Participants indicated how often they engaged in the following behaviors: getting physical exercise, spending time outdoors in nature, spending time on screens or devices (e.g., phone, video games, TV), eating home-cooked meals, eating takeout/delivered food, going to the grocery store, going to farmer’s markets, using farm shares or community-supported agriculture, and using online grocery shopping/grocery delivery, relative to their behaviors pre-pandemic. For these questions, we modified the original response scale, so that participants indicated whether they engaged in each behavior: more often, less often, no change, or N/A. We began with behaviors from the ECHO questionnaire and then added some additional behaviors of interest, specific to food acquisition (e.g., use of online grocery shopping, farmer’s markets).

#### 2.3.3. Current Food Acquisition and Eating Behaviors

Parents were asked how frequently meals were prepared at home during the past two months with response options including 0–1 times per week, 2–3 times, 4–5 times, and more than 5 times per week [8]. Restaurant use was assessed by asking parents how often their child ate food from restaurants during the past two months in three different contexts: dine-in, take-out, or delivery. Response options included: never, once a month or less, 2–3 times a month, once a week, 2–3 times a week, and 4 or more times a week. Parents were also asked to indicate how safe they felt it was to obtain food from a restaurant as dine-in, take-out, or delivery (response options included: very unsafe, somewhat unsafe, somewhat safe, very safe). For each mode of restaurant use, parents also reported typical behaviors over the past 2 months, including who typically selected the child’s restaurant meal (the child, the responding parent, the responding parent and child together, another adult, or another adult and the child together) and the most important (1) to least important (7) reasons for the child’s typical restaurant meal selection (taste, habit, cost, nutrition, appeal, treat, something new). These items were generally administered verbatim from previous restaurant research [30], with exceptions being the aforementioned change to the time frame of interest (i.e., the past two months), as well as asking participants to rank order the reasons for the child’s meal choice rather than having them select all reasons that applied. In addition, the perceived restaurant safety item was newly developed for the present study.

Three additional restaurant-related items were developed for the present study. Parents reported what was typically ordered for the child when ordering restaurant food for take-out or delivery: their own kid’s meal, their own adult meal, shared food with other family members, or other. Parents also ranked their top five reasons for deciding to order restaurant food for take-out or delivery and the top five reasons affecting their choice of restaurant from a list of options (e.g., no time, no groceries, cost, promotion, treat for child, treat for self, appeal, taste, nutrition, something new, habit, convenience, technology, support for restaurants).

### 2.4. Data Analysis

Frequencies (for categorical variables) and means and standard errors (for continuous variables) were calculated for demographic/contextual variables. Items from the PSS-4 were reverse-scored as appropriate and then summed, resulting in a composite score ranging from 0–16, with higher scores indicating higher stress. Frequencies (for categorical variables) and means and standard errors (for continuous variables) were calculated to describe: (1) parent-reported impacts of COVID-19 on various aspects of daily life and energy-balance-related behaviors, including employment, child care and instruction, physical activity, screen time, grocery shopping, cooking, and ordering take-out or delivery; as well as (2) families’ current food acquisition and eating behaviors. These included: frequency of preparation of meals at home, children’s consumption of restaurant foods (frequency of dining in, take-out, and delivery), perceived safety of restaurant foods, who chose children’s restaurant meals and types of meals chosen, and factors affecting children’s meal choices and restaurant choices. Analysis of factors affecting children’s meal choices were restricted to parents who reported playing a role in deciding the child’s meal order (determining it themselves or with the child), as those not playing any role in the decision would not be expected to know which factors contributed to the decision. 

Given the potential for differential impacts of COVID-19, we also explored whether current food acquisition behaviors differed by sociodemographics. We tested models that considered parent age, sex, race/ethnicity, education, and family income as predictors of: the frequency of home cooking, dining out, take-out, and delivery, as well as the perceived safety of each mode of restaurant use. Linear regression models were used unless distributions of residuals violated normality assumptions, in which case outcomes were dichotomized, with logistic regression used to predict meaningful outcomes (e.g., rating restaurant dining as safe vs. unsafe). Backwards deletion was used to arrive at final models, retaining independent variables that predicted outcomes at *p* < 0.05. All analyses incorporated sampling weights, so that results were representative of U.S. parents with 4- to 8-year-old children. Sampling weights were based on parent age, sex, race and ethnicity, education, income, region, marital status, household size, and number of children under 18 years. Data were analyzed using SAS 9.4 (Cary, NC, USA).

## 3. Results

### 3.1. Participant Demographics, Characteristics, and Context

A majority of responding parents were married or living with their partner (83%), with a median of 2 children in the household. Most parents (95%) reported being a primary caregiver of the target child, indicated by reporting that they were the parent or guardian in the household who is the most familiar with the child’s daily activities, or that they and another parent or guardian were equally familiar. Despite variability in family income, reported food insecurity was very common among families over the past two months. More than two-thirds (69%) of parents met the criteria for food insecurity by reporting that they had often or sometimes felt that they might run out of food and not have money to purchase more (66%); or that it was often or sometimes true that the food they had purchased hadn’t lasted and they didn’t have money to purchase more (56%). 

In terms of current COVID-related protection measures, a majority of parents reported that masks were mandated in the city or town where they resided (90%), and that in most cases, at least some closures had been reversed, with very few (38%) or some (44%) businesses closed at the time of survey administration. About half of parents reported that restaurants were open for both indoor and outdoor dining at a reduced capacity, while approximately one-third (29%) reported restaurants in their town or city could only offer take-out or delivery. In many cases, changes to children’s schooling persisted: parents reported that 47% of children were attending school virtually, and 31% were being homeschooled, while 21% attended in-person elementary school, and 19% received care outside the home (including preschools, after-school programs, and child-care centers). Parents reported an average stress level of 7.2 (standard error of the mean (SEM) = 0.14) on the PSS-4′s 0–16 scale. Additional demographic and contextual variables are reported in Table 1.

### 3.2. Daily Life Changes during the COVID-19 Pandemic 

Parents reported changes to employment as a result of the COVID-19 pandemic, including that one-third (38%) moved to working remotely (from home) while 14% lost their job permanently. One-third (30%) of parents also indicated that their job put them at an increased risk for getting COVID-19. Parent-reported impacts on employment appear in Table 2. The pandemic impacted daily home life such that a majority of parents reported either themselves, another person in the home, or a combination of the two had to take over teaching or instruction of the child, and a substantial proportion of parents (48%) reported spending more quality time with children than they had prior to COVID-19. Table 3 illustrates further parent-reported impacts of COVID-19 on child care and schooling and care for others in the household.

Parent-reported impacts of COVID-19 on energy-balance-related behaviors—physical activity, screen time, grocery shopping, cooking, and ordering take-out and delivery—appear in Table 4. Two-thirds of parents (66%) reported spending more time on screens or devices while only a small percentage (13%) reported less time spent on screens or devices. Parent-reported impacts on time spent exercising and in nature were more varied, with substantial percentages of respondents each reporting increases and decreases in these behaviors (Table 4). A majority of parents reported eating home-cooked meals more often than before the pandemic (64%). Almost half of parents (44%) reported eating take-out and delivery less often, while 22% reported no change from before the pandemic. Half of parents (48%) reported going to the grocery store less often than they did pre-pandemic, with about one-quarter (26%) indicating increased frequency. About half of parents (49%) reported increases in use of online grocery shopping. 

### 3.3. Food Acquisition and Eating Behaviors

Recent food acquisition and eating behaviors are reported in Table 5. Almost three-quarters of parents (71%) reported preparing meals at home at least four days per week during the last two months. Parent-reported frequency of dining-in at restaurants by children during that same period revealed that around one-quarter (27%) were dining-in at least once per week, and around one-third had restaurant food via take-out (37%) and delivery (34%) at least once per week. Just under half of parents (47%) reported that they felt dining-in at a restaurant was either very safe or somewhat safe while take-out and delivery were reported to be either somewhat safe or very safe by approximately three-quarters of parents. Over half of parents were involved in deciding what their child ordered to eat from restaurants, either making the decision on their own or together with their child. Parents reported that when ordering take-out or delivery, over three-quarters of children (77%) had their own children’s meal, while one-quarter of children had their own adult meal (26%), and one-third (31%) shared food with other family members (selecting all options that applied). When it came to ranking reasons for choosing the child’s meal, on average, parents who had a hand in the decision (46.7%) reported that taste was the most important reason, followed closely by nutrition (Table 6). When asked about reasons for deciding to order take-out or delivery, the most popular selections were convenience and taste. Table 7 displays all reasons for take-out and delivery choices.

In terms of individual differences in current food acquisition behaviors, parent gender was the only significant predictor of cooking at home in the tested multivariable models, with female parents cooking at home more than other respondents (t = 2.6, *p* < 0.01). Being a female parent also predicted a lower frequency of the child consuming food at restaurants (t = −3.1, *p* < 0.01), as did lower income (t = 4.5, *p* < 0.0001; R^2^ for final multivariable model = 0.08). Parent education was the only significant predictor of children’s frequency of take-out consumption in the tested multivariable models, such that parents with lower education levels reported less take-out (t = 5.9, *p* < 0.0001; R^2^ = 0.05). Lower parent education was also linked with less restaurant delivery (t = 2.52, *p* < 0.05), as were lower income (t = 2.60, *p* < 0.01) and non-Hispanic Asian race (t = 2.01, *p* < 0.05; R^2^ = 0.09). Race and ethnicity was the main predictor of variability in the reported safety of restaurant use: using dummy-coded indicators of race/ethnicity, Hispanic (t = −4.3, *p* < 0.0001), non-Hispanic Black (t = −5.4, *p* < 0.0001), and non-Hispanic Asian (t = −3.0, *p* < 0.01) respondents were all less likely to view in-person dining as safe, as were respondents with lower education (t = 2.3, *p* < 0.05). Hispanic respondents were less likely than respondents in other race/ethnicity categories to report that restaurant delivery was safe as well (t = −2.4, *p* < 0.05). Other demographic factors were unrelated to perceived restaurant safety.

## 4. Discussion

The COVID-19 pandemic is changing the way families in the U.S. eat, work, and live. Such periods of drastic flux can potentially lead to the establishment of new behaviors and habits, for better or worse. Evidence of short-term impacts of COVID-19 on families’ energy-balance-related behaviors has emerged, but to date, little is known about potential longer-term impacts. The present nationally representative survey of U.S. parents was administered in Fall 2020, a time during which many initial protection measures and restrictions had been lifted, thus offering a glimpse into families’ potential “new normal”. Results suggest there may be some longer-lasting impacts of COVID-19, including on children’s schooling and time spent at home, as well as the reported prevalence of food insecurity, which remains high. Families reported increased home food preparation while still using restaurants a few times each month. Take-out and delivery were perceived as safer than dining in and were used at higher rates, with some sociodemographic differences in restaurant use and perceived safety. Understanding families’ behaviors during this phase of the pandemic can provide insight into potential lasting impacts on families’ lifestyles and health and can inform future research and policy.

Surveys of families in many nations have contributed evidence of short-term impacts of COVID-19 on energy-balance-related behaviors. For example, changes in the home food environment and increases in food insecurity were reported in a survey study of 584 U.S. parents of children ages 5 to 18 [31], and in a survey study of 254 families with young children in Ontario, Canada, substantial percentages of parent respondents reported eating more snack foods, eating less fast food or take-out food, spending more time cooking, and eating more meals with their children [32]. Both of these surveys were fielded in April and May 2020, and these apparent short-term impacts may be tied to broader contextual changes, including COVID-19-related changes to work and family life. 

In the present study, parents’ self-reported impacts of COVID-19 revealed that they were working from home more and taking greater part in the care and instruction of their children, with many reporting reduced work hours. Notably, more than six months into the pandemic, the majority of respondents’ children were either attending school virtually or being homeschooled. In 2019, only 3% of children were homeschooled while the remaining 97% of the 50 million children enrolled in primary or secondary education in the U.S. attended school in person, highlighting an ongoing, drastic change to family routines [33]. It is not surprising that levels of reported stress were higher than previously-established norms [34,35], consistent with findings from other nations that reflect high levels of stress during COVID-19 [32,36]. High stress may impact decision-making, including decisions about energy-balance related behaviors, as research supports the idea that stress can catalyze a change from analytical to intuitive decision-making [37]. Under ongoing high levels of stress, food-related decisions may be governed by heuristics (i.e., short-cuts, such as: what is the habitual, automatic, or convenient choice?) or emotions (what will make me feel better?). In addition to potential links with stress, persisting changes in childcare and schooling may be linked to energy-balance-related variables reported herein, as children spending more time at home could impact food security, acquisition, and/or preparation.

The present results suggest that high levels of food insecurity persist among families more than six months into the pandemic. Over two-thirds of parents indicated they had experienced some form of food insecurity in the previous two months. While the current 2-item food insecurity screening tool may be limited compared to longer measures, the general point that very high proportions of families are suffering from food insecurity during COVID-19 is consistent with other research, including prior findings that more than half of surveyed families reported some form of food insecurity during COVID-19, a significant increase from prior to the pandemic [31]. Persistent food insecurity has implications for child health, as food insecurity has been linked with poor dietary intake and unhealthy weight outcomes [15]. During the initial months of the pandemic, increases in snacking behavior and low nutrient quality foods were seen [7,15]. It has also been well documented that many children in the U.S. already ate few nutrient dense foods, like fruits and vegetables, prior to the pandemic and ate at restaurants frequently [17,18]. Mitigation of food insecurity is an important research and policy target. 

While families juggle ongoing contextual challenges, parents reported acquiring food from various sources. Similar to earlier studies, the majority of parents in the present nationally-representative sample reported more home cooking, with a substantial percentage also reporting less restaurant take-out and delivery as a result of the pandemic. Early reports stated that restaurant purchasing was down 30% in March 2020 compared to the previous year, and another study reported that in the early months of the pandemic, people were avoiding restaurants completely [13,25]. Yet the present results do suggest that restaurant dining may be moving toward pre-pandemic levels for many families. Overall reported frequencies of restaurant use showed that around half of children were eating restaurant food at least a few times each month via in-person, take-out, and delivery, in the context of two-third of families reporting that in-person dining was currently available in their area. Studies examining children’s frequency of eating at restaurants in-person or via take-out from years prior to the COVID-19 pandemic show similar patterns (e.g., 56% of children ate at restaurants at least 2–3 times per month) [30]. In particular it appears higher-income, higher-education families are more likely to be returning to pre-COVID-19 restaurant habits.

However, there is a subset of parents who do not view restaurant dining as safe, with the present results indicating that perceived safety is lower among parents with lower education and among Hispanic, non-Hispanic Black, and non-Hispanic Asian parents. These differing perspectives could reflect different sources of information (e.g., engagement with varying types of media) and/or varying lived experiences as different demographic groups may occupy different spaces (e.g., with variable mitigation measures in place, variable levels of exposure among essential workers). Generally, a larger percentage of parents in the present study viewed take-out and delivery as safe, compared to dining-in, highlighting a need for more research on individual differences, as well as on these understudied modes of restaurant dining in the future. 

Convenience and taste were the primary reported reasons for take-out and delivery choices, and taste and nutrition were highly ranked reasons for child meal choices from restaurants overall. Surprisingly, cost was not one of the higher-ranked choices for children’s meal choices. Research prior to COVID-19 has shown that for many low-income families, cost is a dominant factor in meal selection, even after the decision to eat out is reached [38], while in higher socioeconomic status families, taste and cost are both important [39]. The current findings are consistent with prior research showing that liking and taste are important factors for children’s meal selection [30]. Perhaps in the context of the COVID-19 pandemic, families may be particularly motivated to seek convenient options and provide their child with a tasty meal when they do decide to dine out. 

Limitations of the present study include the use of a self-report measure, which can be subject to social desirability bias. Minor modifications to some existing survey items were made to fit with the aims of the present study (e.g., changing the time frame to the past two months). Furthermore, the short versions of some survey instruments were used (i.e., food insecurity, perceived stress). While demonstrations of the validity of these short forms are present in the literature, the long-form versions of these measures generally have superior psychometrics. In the present study, the PSS-4 had a relatively low Cronbach’s alpha, as observed in some prior research [40], suggesting that longer versions of the Perceived Stress Scale may be preferable when feasible. A strength of the study is the use of sampling weights to achieve a nationally representative sample of families with 4- to 8-year-old children in the U.S. The study was conducted in October of 2020, more than six months after the pandemic was recognized. Evidence of shorter-term impacts of the pandemic on energy-balance-related behaviors and daily life may reflect the novel nature of the pandemic and stringent initial lockdowns. As the pandemic continues, and restrictions have relaxed in many areas, it is possible that families may show some returns to pre-pandemic lifestyles, while other changes may be longer-lasting. Results from the present study illustrate ongoing impacts on children’s schooling and food insecurity, with a possible return to pre-pandemic behaviors in other areas, such as restaurant use, among many families. These findings have implications for future energy balance-related intervention research, as such work must take place in the context of families’ realities. For example, current healthy eating interventions in restaurants would need to be feasible and acceptable in the context of families’ varied use of and perceived safety of restaurant dining. 

Continued surveillance can elucidate lasting shifts in behavior as a result of the pandemic and guide interventions to be safe, feasible, and relevant in the future. Nationally representative studies allow for generalizable conclusions, and examination of individual differences can facilitate targeted research and policy efforts to promote health and provide support to those most impacted. Research and policy efforts to address food insecurity are also of paramount importance.

## 5. Conclusions

Key goals of the present study were to describe parent-reported impacts of COVID-19 and current family food acquisition and eating behaviors more than six months into the pandemic. Results suggest continuing impacts of COVID-19 in many areas (e.g., schooling at home, food insecurity) and a potential move toward normalcy in other areas (e.g., restaurant dining) among some families, with some evidence of sociodemographic differences. Most parents reported that their child was consuming food from restaurants at least 2–3 times per month, with lower use of restaurants and lower reported perceived safety among some sociodemographic groups. These observations have implications for future research as the pandemic progresses, as well as intervention and policy efforts as scientists and policymakers consider the best ways to improve health and well-being among families in the COVID-19 era.

## Figures and Tables

**Table 1:** Participant characteristics for study sample (*n* = 1000) (weighted frequencies and means).

Sociodemographic Variables	%	Mean (SEM)	Sociodemographic Variables Cont.	%	Mean (SEM)
**Gender**			**Race/ethnicity**		
Female	55		White	69	
Male	45		Black or African American	12	
Transgender	1		Asian	11	
Other	0		Other	8	
**Marital status**			Hispanic	22	
Now Married/Living with Partner	83		**Highest level of education completed**		
Single/Never Married	9		≤High School/GED ^a^	20	
Divorced/Separated/Widowed	8		Some college/Tech/Associates	37	
**Age (years)**		38.8 (9.5)	Bachelor’s Degree	17	
18–24	2		≥Graduate Degree	26	
25–34	31		**Government benefits received at any point in 2020**		
35–44	45		SNAP ^b^	42	
45–54	14		WIC ^c^	30	
55+	8		Medicaid	46	
**Body mass index**			Disability	21	
25.0–29.9 (overweight)	33		TANF ^d^	25	
≥30.0 (obese)	17		**Number of children in the household**	2.4 (1.2)	
**Current employment status**			1	18	
Employed Full Time	63		2	45	
Employed Part Time	6		3	24	
Self-employed	6		4+	12	
Not employed	7		**4–8-year-old child with most recent birthday**		
Homemaker/Stay-at-home	14		Age (years)		6.2 (1.4)
**Household income (per year)**			Gender—% Male	55	
<$24,999	10		Gender—% Female	45	
$25,000–$34,999	7		**Child eligible for free or reduced-price school meals (*n* = 593 due to age range)**		
$35,000–$49,999	11				
$50,000–$74,999	16		Yes	59	
$75,000–$99,999	15		No	31	
>$100,000	41		Don’t know	9	
**Coronavirus disease 2019 (COVID-19)-related restrictions in place in parent’s town or city**					
**General restrictions**			**Restaurant restrictions**		
Most businesses are closed	18		Can offer take-out or delivery	29	
Some businesses are closed	44		Can dine in: Outdoors only	15	
Very few businesses are closed	38		Both outdoors and indoors: reduced capacity	50	
**Masks mandated (public places, indoors)**			Both outdoors and indoors: full capacity	6	
Yes	90				

^a^ GED – General Educational Development Test. ^b^ SNAP—Supplemental Nutrition Assistance Program. ^c^ WIC—Special Supplemental Nutrition Program for Women, Infants, and Children (31). ^d^ TANF—Temporary Assistance for Needy Families.

**Table 2:** Parent-reported impacts of the COVID-19 pandemic on employment (*n* = 1000).

Employment Changes	Yes (%)	No (%)
Moved to working remotely/from home	38	62
Permanent job loss	14	86
Temporary job loss	20	80
Got a new job	14	86
Reduced work hours	38	62
Increased work hours	25	75
Laid off employees	16	84
Work was affected in some other way	39	61
Put at increased risk for getting COVID-19	30	70
Did not have a paying job before COVID-19	10	90

**Table 3:** Parent-reported impacts of the COVID-19 pandemic on child-care, schooling, and care of other family members (*n* = 1000).

Family Care Changes	Yes (Me) (%)	Yes (Person in Household) (%)	Yes (Me & Person in Household) (%)	No (%)	N/A (%)
Took over teaching/instruction of child	47	16	20	12	4
Child care or babysitting unavailable	25	14	13	30	18
More quality time with child(ren)	48	14	29	7	1
More time spent caring for other family members	38	15	17	23	7

**Table 4:** Parent-reported impacts of COVID-19 on physical activity, outdoor time, screen time, food acquisition, and cooking (*n* = 1000).

Lifestyle Behavior Changes	More Often	Less Often	No Change	N/A
	(%)	(%)	(%)	(%)
Physical Exercise	40	29	27	4
Time spent: outdoors/in nature	36	40	22	2
Time spent: on screens/devices	66	13	19	2
Eat home-cooked meals	64	12	23	1
Eat take-out/delivery	33	44	22	1
Go to the grocery store	26	48	25	2
Go to farmer’s markets	20	34	21	25
Use of farm shares or community supported agriculture	19	23	22	36
Used online grocery shopping	49	11	19	20

**Table 5:** Parent-reported restaurant use and ordering for their 4- to 8-year-old-child and perceived restaurant safety (*n* = 1000 *).

Frequency	How Often Child Ate at/from Restaurants (Past 2 Months)		
	In-person (%)	Take-out (%)	Delivery (%)
Never	28	14	23
<1×/month	24	24	20
2–3×/month	21	25	23
1×/week	13	19	15
2–3×/week	11	14	15
>4/week	3	4	4
	**Who Typically Decided What to Order for Child (Past 2 Months)?**		
	In-person (%)	Take-out (%)	Delivery (%)
Mother (Reporting Parent)	12	16	18
Father (Reporting Parent)	17	16	18
The child	33	28	23
Parent and child together	28	34	34
Another adult	5	4	4
Child and another adult	4	2	3
	**How Safe Do You Currently Feel That It Is to:**		
	Dine indoors at a restaurant (%)	Eat take-out food (%)	Eat delivered food (%)
Very unsafe	21	9	6
Somewhat unsafe	32	16	16
Somewhat safe	29	45	46
Very safe	18	29	31

* All 1000 parents responded to restaurant frequency questions (for dining in-person, take-out, and delivery). Parents who responded “never” to these were not asked to respond to the subsequent questions about that mode of restaurant use.

**Table 6:** Parent ranking of the importance of different factors when choosing a restaurant meal for their child over the past 2 months (*n* = 467) ^a,b^.

Reasons for Meal Choice	Dining in-Person	Ordering Take-Out	Ordering Delivery
	Mean (SEM)	Mean (SEM)	Mean (SEM)
Taste—child likes the foods in the meal	3.2 (0.1)	3.2 (0.1)	3.3 (0.1)
Habit—what the child typically orders	3.7 (0.1)	3.6 (0.1)	4.1 (0.1)
Cost—price of the meal	4.6 (0.1)	4.5 (0.1)	4.5 (0.1)
Nutrition—health of the meal	3.5 (0.1)	3.6 (0.1)	3.8 (0.1)
Appeal—the meal looks good	4.2 (0.1)	4.2 (0.1)	4.0 (0.1)
Treat—my child doesn’t get it often	4.3 (0.1)	4.1 (0.1)	4.0 (0.1)
New—trying a new flavor	4.5 (0.1)	4.7 (0.1)	4.5 (0.1)

Parents ranked factors shown above on a 7-point scale, where 1 was the most important reason, and 7 was least important. The means depict the average rank for each reason, with lower means indicating that the reason was more important on average. ^a^ All 1000 parents responded to restaurant frequency questions (for dining in-person, take-out, and delivery). Parents who responded “never” to these were not asked to respond to the subsequent questions about that mode of restaurant use. ^b^ In addition, only parents who reported playing a role in deciding the child’s meal order (determining it themselves or with the child) were included in this analysis, as those not playing any role in the decision would not be expected to know which factors contributed to the decision.

**Table 7:** Reasons behind parents’ take-out and delivery orders over the past 2 months.

Reasons for Take-Out/Delivery	Participants Selecting This Reason (%) ^a^	Mean (SEM)
Convenience: it was fast/easy	50.1	2.7 (0.1)
Taste: the meal would taste good	43.1	3.1 (0.1)
Treat self: to treat myself	41.6	3.0 (0.1)
Treat child: to treat my child(ren)	41.8	3.1 (0.1)
No time: I wanted to save time/didn’t have time to cook	40.5	2.5 (0.1)
Promotion: because of a special such as a discount	29.3	3.3 (0.1)
Support: trying to support restaurants that my family likes	28.7	3.0 (0.1)
Cost: because of its price	27.1	3.1 (0.1)
Appeal: the meal looked good in the picture on the menu/website	26.2	3.3 (0.1)
Something new: trying new foods/flavors	26.0	3.3 (0.1)
Habit: it is what I usually do	25.5	3.2 (0.1)
Nutrition: the meal would be healthy	24.7	2.9 (0.1)
Technology: Able to order using an online delivery service	23.6	3.0 (0.1)
No groceries: did not have groceries or ingredients to cook	22.2	2.8 (0.1)
**Reasons for restaurant choice**		
Taste: my family likes the food from that restaurant	59.4	2.8 (0.1)
Convenience: it is fast/easy	53.7	2.8 (0.1)
Cost: because of its price	47.0	3.0 (0.1)
Treat: it is a treat that my family does not get often	44.6	3.1 (0.1)
Habit: it is a restaurant we usually order from	41.1	3.1 (0.1)
Promotion: because of a special such as a discount	37.7	3.1 (0.1)
Support: trying to support restaurants that my family likes	36.2	3.1 (0.1)
Appeal: the food at that restaurant looked good in the picture	35.5	3.3 (0.1)
Nutrition: because it offers healthy food	34.3	3.0 (0.1)
Technology: able to order using an online delivery service	33.4	3.1 (0.1)
Something new: trying a new flavor	27.2	3.1 (0.1)

^a^ Here, parents ranked their top 5 reasons in each category. The (weighted) percent of parents endorsing each option is shown, as well as the average rank order for each option among parents endorsing it.

## Data Availability

Access to study data can be provided by the corresponding author upon reasonable request after the study team has completed planned data analyses.

## Abbreviations

U.S.	United States
PSS-4	Perceived Stress Scale: 4-item version
EPII	The Epidemic—Pandemic Impacts Inventory
ECHO	Environment Influences on Child Health Outcomes
